# Protracted outbreak of *S*. Enteritidis PT 21c in a large Hamburg nursing home

**DOI:** 10.1186/1471-2458-7-243

**Published:** 2007-09-13

**Authors:** Christina Frank, Udo Buchholz, Monika Maaß, Arthur Schröder, Karl-Hans Bracht, Paul-Gerhard Domke, Wolfgang Rabsch, Gerhard Fell

**Affiliations:** 1 Department for Infectious Disease Epidemiology, Robert Koch Institute, Berlin, Germany; 2 Institute for Hygiene and the Environment, Hamburg, Germany; 3 District Public Health Authority, Hamburg-Wandsbek, Germany; 4 Consumer Protection Authority, District Stormarn, Germany; 5 National Reference Centre for Salmonella and other Enteric Pathogens, Robert Koch Institute, Wernigerode, Germany

## Abstract

**Background:**

During August 2006, a protracted outbreak of *Salmonella *(*S*.) Enteritidis infections in a large Hamburg nursing home was investigated.

**Methods:**

A site visit of the home was conducted and food suppliers' premises tested for *Salmonella*. Among nursing home residents a cohort study was carried out focusing on foods consumed in the three days before the first part of the outbreak. Instead of relying on residents' memory, data from the home's patient food ordering system was used as exposure data. S. Enteritidis isolates from patients and suspected food vehicles were phage typed and compared.

**Results:**

Within a population of 822 nursing home residents, 94 case patients among residents (1 fatality) and 17 among staff members were counted 6 through 29 August. The outbreak peaked 7 through 9 August, two days after a spell of very warm summer weather. S. Enteritidis was consistently recovered from patients' stools throughout the outbreak. Among the food items served during 5 through 7 August, the cohort study pointed to afternoon cake on all three days as potential risk factors for disease. Investigation of the bakery supplying the cake yielded S. Enteritidis from cakes sampled 31 August. Comparison of the isolates by phage typing demonstrated both isolates from patients and the cake to be the exceedingly rare phage type 21c.

**Conclusion:**

Cake (various types served on various days) contaminated with *S*. Enteritidis were the likely vehicle of the outbreak in the nursing home. While the cakes were probably contaminated with low pathogen dose throughout the outbreak period, high ambient summer temperatures and failure to keep the cake refrigerated led to high pathogen dose in cake on some days and in some of the housing units. This would explain the initial peak of cases, but also the drawn out nature of the outbreak with cases until the end of August. Suggestions are made to nursing homes, aiding in outbreak prevention. Early outbreak detection is crucial, such that counter measures can be swift and drawn-out outbreaks of nosocomial food-borne infections avoided.

## Background

In early August 2006 a large senior care facility in Hamburg reported a nosocomial outbreak of gastroenteritis to the local health department. Residents were falling ill with symptoms of diarrhoea and vomiting and the outbreak was continuing well beyond one week. Staff were also affected. Stool samples from early cases yielded *Salmonella enterica *ssp.*enterica *serotype Enteridis (*S*. Enteritidis). This is the *Salmonella *serotype most frequently reported in Germany (30,000–50,000 cases per year), especially in later summer. As elsewhere, poultry and poultry products like eggs are considered the most important food vehicles for *S*. Enteritidis infections [1,2]. On 19 August a formal investigation into the protracted outbreak was launched, aiming to identify and contain the apparently continuous source of infection.

## Methods

An extensive site visit at the senior care facility was conducted by a multidisciplinary team (local health administrators, food safety inspectors, epidemiologists) in order to spot potential mechanisms of transmission and to examine kitchens and the food distribution system. Kitchens were scrutinized for hygiene problems, and all available reference food samples were tested. Kitchen personnel were asked to provide stool samples which were investigated for *Salmonella*. Premises of a suspected food supplier were also investigated: environmental swabs, employee stools, raw products and current product samples were tested for *Salmonella*. A number of *S*. Enteritidis isolates from the outbreak were submitted to phage typing (method according to Ward [3], extended scheme) and compared to other isolates currently circulating in Germany by the National Reference Centre for *Salmonellae *(NRC).

A cohort study among the residents to identify potential food vehicles among the residents focused on the early part of the outbreak (dates of onset between 6–10 August) and was conducted in two of the larger among the nine nursing units (units 1 and 9) strongly affected by the outbreak. It was later extended to four units (data on selected variables collected on units 2 and 4). The outbreak period was defined to begin on the date of onset of the first case to be recognized after two incubation periods (six days) without cases, and to end with the date of onset of the last case to be recognized before two incubation periods without new cases. The case definition was "diarrhoea and/or vomiting on any day between 31 July and 4 September". Patients with diarrhoea and/or vomiting but *S*. Enteritidis-negative stool samples were excluded, patients whos' stool was not tested were included as probable cases and analysed together with the laboratory confirmed cases. The total number of persons tested for *Salmonella *in association with the outbreak remains unknown, as medical care both for residents and staff lies in the hands of each person's individual GP.

Preliminary interviews indicated that residents were unlikely to remember correctly what they ate two weeks previously. Thus instead of information from interviews, data from the central kitchen's computerized patient food ordering system and from the peripheral kitchens' sets of note cards (individual standing orders for breakfast and dinner sandwiches) were used. Data analysis was conducted in SPSS^® ^Version 13.0. The attack rate (AR) was defined as the proportion of residents matching the case definition among the population in question. Based on the AR, relative risks (RR) for individual exposures and their defining 95% confidence intervals (95% CI) were calculated. Elevated RR were tested for the influence of confounding or interactions by stratified and multivariate analyses. The attributable fraction was calculated according to the following formula: AF = [(RR-1)/RR] × [(# case patients exposed)/(# case patients)].

## Results

### Case numbers and case specifications

**Figure 1:**
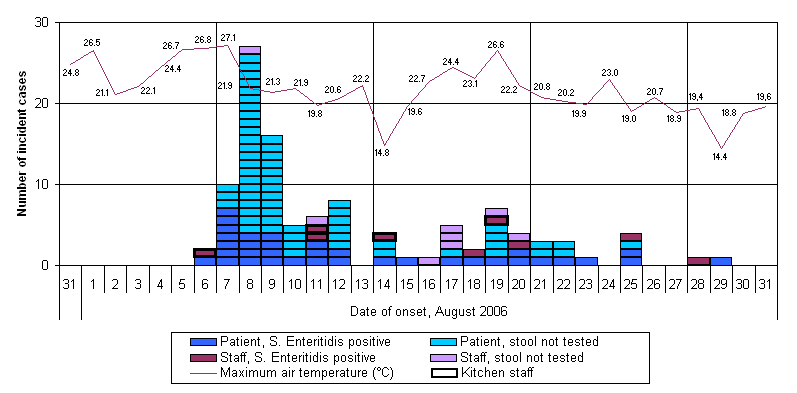
Epi curve of the outbreak (n = 94 patients and 17 staff), including information on Hamburg daytime maximum air temperature (Source: German Meteorological Service, DWD).

No data on the background incidence for diarrhoea and vomiting were available for the senior care facility. The epidemic curve shows a first peak of cases (n = 53) between 7 and 9 August followed by approximately two weeks with zero to eight cases daily (figure 1). The outbreak lasted for 24 days.

The entire population of the complex is approximately 1200 inhabitants. A total of 822 persons live in nine nursing units, cared for by 330 staff. The remainder resides in eight houses for assisted living. Only inhabitants of the nursing units ("residents") were affected by the outbreak, albeit with different AR (table 1). Only unit 8, caring for persons with dementia, remained unaffected. Unit-specific attack rates over the entire duration of the outbreak ranged from 0 to 23.2% (median of 13.9%). Altogether 94 residents and 17 staff members were counted as cases between 6 and 29 August. One of the residents died in consequence of salmonellosis (case fatality ratio among nursing home residents: 1.1%). Fifteen (88%) of 17 cases among staff had dates of onset after 10 August, while 58 (61%) of 94 cases among residents occurred before 11 August. The initial peak of cases lagged by two days behind a period of three very warm summer days (daytime high temperatures around 27°C) in early August, which would have increased temperature in the non-air-conditioned resident rooms with large windows significantly.

**Table 1:** Case numbers and attack rates (AR) per nursing unit

Unit	No. Inhabitants	No. affected overall	AR overall (%)	No. affected 6–10 August	AR 6–10 August (%)	No. staff affected overall
1	116	20	17.2	12	10.3	
2	118	18	15.3	10	8.5	1
3	114	11	9.7	7	6.1	1
4	79	11	13.9	9	11.4	3
5	97	6	6.2	0	0.0	3
6	56	13	23.2	7	12.5	
7	41	5	12.2	1	2.4	
8 (dementia pts)	136	0	0.0	0	0.0	1
9	65	14	21.5	7	10.8	1
Kitchens						5
Others						2
Totals	822	98	11.9	53	6.5	17

### Kitchens and food delivery system

**Figure 2:**
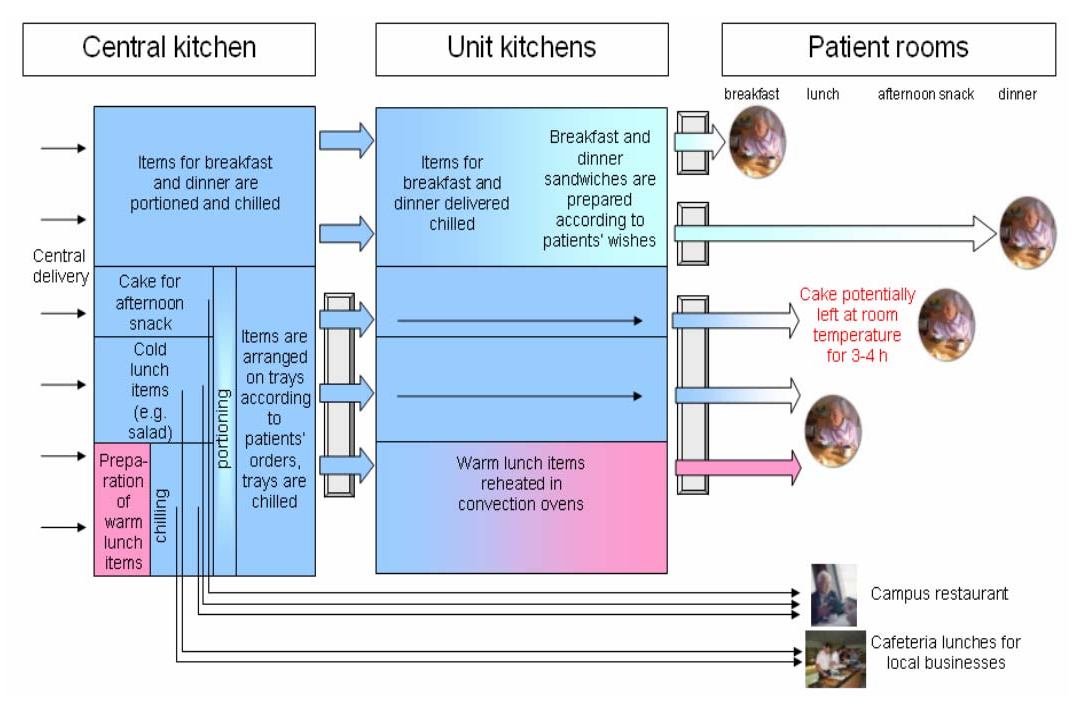
Food distribution system within the nursing units and other customers of the central kitchen.

The central kitchen functioned as a clearing house for all meals served in the nursing units (for details see figure 2). Cold food items for breakfast and dinner arrived in small lots in the unit kitchens and were used there to prepare breakfast and dinner, served on trays according to residents' standing orders. Warm meals were prepared in the central kitchen, chilled, portioned and arranged on trays according to residents' orders from a weekly menu. Items for warm consumption were reheated in the unit kitchens utilizing cook-and-chill procedures.

Baked goods were delivered to the central kitchen daily by a baker from a county outside of Hamburg. For the daily afternoon snack various types of cake were delivered by the baker on large rectangular baking sheets and portioned in the central kitchen. On Sundays layered cream cake was customary, fruit-type cakes during the week. The same type of cake distributed by the central kitchen was delivered daily in classical round shape to the on-campus-restaurant, through which the assisted living seniors not affected by the outbreak had access to central kitchen lunch and afternoon-snacks. The kitchen also provided lunches (but not cake) to a small number of businesses in the area. No complaints about diarrhoeal illnesses were received from these customers.

Unit nursing staff distributed food into residents' rooms for breakfast, lunch and dinner, including the cake on the lunch tray. Staff members were supposed to deposit the "vulnerable" layered cream cake directly into the small patient room refrigerators. Time of consumption of the cake was up to the residents, except for in unit 8. There the pieces of cake were refrigerated in the unit kitchen until staff took the cake to the individual rooms and assisted residents in eating it.

All reference food samples available (only food items prepared in the central kitchen – items brought in from the outside and just distributed there were not sampled) tested negative for *Salmonella*. Five kitchen employees fell ill with salmonellosis through the course of the outbreak. The employee affected on 6 August worked in the central kitchen but was not involved in cake or light meal processing. Four other kitchen employees fell ill between 11 and 19 August. In addition, three asymptomatically infected staff members tested positive for *S*. Enteritidis. Two of them, one unit kitchen helper (stool positive on 21 August) and a central kitchen chef (stool positive on 22 August) were involved with food handling, the third (stool positive on 29 August) did not handle food.

### Results of the cohort study

Units 1 and 9 housed 181 residents. Of 114 residents with available information on sex 86 (75%) were women. Mean age in men and women was 88 years (range in men 76 – 95 years, range in women 75 – 103 years). Nineteen (10.5%) residents were case-patients with symptom onset before 11 August. Units 2 and 4 housed 197 residents (83% women). Mean age was 87 years with a range from 66–104 years. Twentythree (11.7%) residents were case-patients with onset before 11 August. Thus, in all four units there were 42 (11.1%) case-patients with symptom onset before 11 August. The only meals or food items that were consistently statistically significant or close to significance were the light meals for lunch and the afternoon cake on 5, 6 and 7 August, respectively (table 2). In units 1 and 9 consumption of cake on Sunday, 6 August, had the highest attributable fraction with 66%, while the maximum attributable fraction of the light lunch meal was only 43% (table 2). There was statistically significant association between the variables light meal on 6 August and afternoon cake consumption on any day (p < 0.01). In an analysis of light meal exposure stratified by cake consumption, the light meal did not remain an independent risk factor (adj. RR MH 1.92, p = 0.72).

**Table 2:** Results of the cohort study: attack rates among those exposed and not-exposed to various food items on 5–7 August, relative risk as measure of association

**Date, meal, food item**	**Exposed**			**Not exposed**			**RR**	**95% CI**	**AF**
					
	**ill**	**not ill**	**AR (%)**	**ill**	**not ill**	**AR (%)**			
Cohort from units 1 and 9, n = 181 (variable denominator due to missing data), only those with RR>2 shown:									
5-Aug, lunch, "light meal"	10	63	15.9	9	118	7.6	2.08	0.89–4.86	28%
5-Aug, afternoon, cake	17	128	13.3	2	53	3.8	3.52	0.84–14.70	64%
6-Aug, lunch, "light meal"	11	47	23.4	8	134	6.0	3.92	**1.68–9.15**	**43%**
6-Aug, afternoon, cake	17	125	13.6	2	56	3.6	3.81	0.91–15.93	66%
6-Aug, dinner, cold cuts	16	116	13.8	3	65	4.6	2.99	0.91–9.87	56%
7-Aug, afternoon, cake	16	125	12.8	3	56	5.47	2.39	0.73–7.87	49%
									
Cohort from units 1, 2, 4 and 9, n = 378 (variable denominator due to missing data), all food items shown:									
5-Aug, afternoon, cake	38	306	13.3	4	70	5.7	2.17	0.80–5.89	49%
6-Aug, afternoon, cake	38	303	13.3	4	69	5.5	2.29	0.84–6.21	51%
7-Aug, afternoon, cake	37	303	12.2	5	68	6.8	1.78	0.73–4.38	39%
6-Aug, lunch, "light meal"	20	123	16.3	22	253	8.7	1.87	**1.06–3.29**	**22%**

AR, attack rate; AF, attributable fraction

### Microbiological results and the investigation of the bakery

No pathogen other than *S*. Enteritidis was cultured from the outbreak case-patients' stools, except for one stool yielding norovirus. All together 45 stools of residents and staff members with diarrhoea and/or vomiting yielded *S*. Enteritidis.

The baker's premises were investigated on 31 August. Two of three types of cake sampled on this day tested positive for *S*. Enteridis. The third cake, various raw products as well as environmental swabs were negative, as were all stool samples from 11 employees collected in subsequent days. Product samples from two different dates in September were also all *Salmonella*-negative. In the county where the baker's premises served local customers, the number of notified *S*. Enteritidis cases rose to 16 in August compared to 1–6 cases in other months of the year 2006. However, in previous years August had also been the peak month with a mean of 18.4 cases. Phage typing is not available for cases notified for routine surveillance purposes.

The 16 human (13 patients and 3 staff members) and 2 cake isolates submitted to phage typing were all phage type (PT) 21c, a rare PT not seen in the NRC for years (table 3), except in an almost concurrent outbreak at an ice cream shop in another state (North Rhine-Westphalia) where vanilla ice cream made on the premises was the vehicle (unpublished data). A connection between the ice cream shop and the baker near Hamburg could not be identified.

**Table 3:** Occurrence of different *S*. Enteritidis phage types from outbreaks in Germany (National Reference Laboratory Surveillance Data)

**Phage type**	**2005 (n = 49 outbreaks)**		**2006 (n = 77 outbreaks)**	
	
	**Number of outbreaks**	**%**	**Number of outbreaks**	**%**
**4**	20	40.8	44	57.1
**8**	6	12.2	11	14.3
**21**	8	16.3	7	9.1
**5**	5	10.2	2	2.6
**1**	3	6.1	3	3.9
**6**	3	6.1	1	1.3
**21c**	0	0.0	2	2.6
**Others**	4	8.2	7	9.1

## Discussion

The outbreak was caused by *S*. Enteritidis PT 21c contaminated bakery products (cake) delivered daily to the nursing home. A combination of the apparent *S*. Enteritidis PT 21c contamination of baked goods from the baker's premises and the findings of the cohort study clearly point to the implicated cakes delivered to the nursing home as the ultimate source of the introduction into the institution. Within the home, both the cakes directly, and likely also cross contaminated other food vehicles and person-to-person transmission served as sources of infection. The association between the consumption of the light meal and cake and the result of a stratified analysis were additional indications that the light meal was no independent risk factor. The meal included a sweet chicken curry with fruits, which may have been particularly attractive to persons who also like cake and sweets in general. It is also possible that the correlation arises from the high proportion of diabetics among the residents, who would have received none or special cake and also may have avoided the sweet curry.

Pathogen dose in the cakes upon delivery likely was low. As suspected in a similar *S*. Enteritidis outbreak in a Spanish hospital [4], nursing home residents may have been particularly vulnerable to develop clinical infection upon ingestion of even a small inoculum. Also, some nursing home residents may have been especially vulnerable to clinical infection due to advanced age and pre-existing illness. Moreover, improper handling and un-chilled storage at the nursing home could have led to various degrees of infectiousness at the time of consumption of the cake, which may explain the absence of additional *S*. Enteritidis cases among the assisted-living inhabitants who ate at the campus restaurant, the unaffected unit 8, or the implicated bakery's local customers. As phage typing is not available for the cases in the bakery's home county, it remains unclear if problems extended to local customers, although given the overall low number of *S*. Enteritidis cases compared to the same month in other years, the problem could not have been significant.

**Figure 3:**
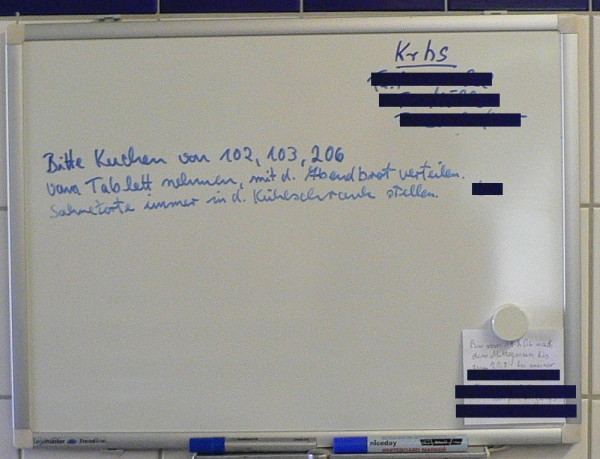
Handwritten sign in the kitchen reminding staff to place layered cream cake in the patient room fridges upon delivery (in German).

During high ambient temperatures in early August, cake – especially layered cream cake served on Sunday 6 August – would have been a very competent food vehicle for *Salmonella *within the nursing units, especially if the cake had been left standing at room temperatures for hours before consumption. The fact that staff were prone to forget depositing the cake into the fridge upon delivery to the room may be indicated by a hand scribbled remainder photographed during the site visit on a unit kitchen white board (figure 3).

Staff members were officially prohibited from sharing residents' meals and should not have eaten or sampled the cake. Except for kitchen staff members having breakfast at the central kitchen, the shift system did not require any staff members to have lunch on the premises. Most brought all their snack food from home and ate major meals elsewhere. The majority of staff affected fell ill after the first big wave of cases among the residents. This may be an indication of spread from case-patients to staff in consequence of nursing care related close contact. Many infected staff members claimed not to have nursed affected residents, however, as specific protection measures were only initiated in the care of recognized case-patients (room-specific gowns, wearing of disposable gloves during all times), asymptomatic infections could have lead to transmission. Person-to-person infection with *salmonella *from nursing home residents to staff has previously been described [5,6], as well as extended outbreaks where infection was carried mainly between patients [7].

As many more residents ate the cake than became case-patients, asymptomatic infections are likely to have occurred. Some employees, both from the central kitchen and from unit kitchens, were affected as well, but apparently were excused from work promptly upon developing symptoms. Since the positive cake samples were taken directly from the baker's premises, nursing home kitchen staff members were likely to have become infected from handling the cake rather than being the source of the outbreak. The kitchen employee with onset on the first day of the outbreak developed symptoms in the evening after work and did not return to work the next day. His only contact to the implicated food items would have been that he possibly consumed them. It is feasible, that during the portioning of the cake onto individual plates in the central kitchen cross contamination of other food items occurred, which may explain some of the other food items associated with an increased risk of developing salmonellosis.

*Salmonella enterica *serotype Enteritidis is the predominant *Salmonella *serovar identified by national reference laboratories in Western Europe. It is important that routine phage typing is carried out such that outbreaks can be recognised and investigated, and trends can be identified. Approximately half of the *S*. Enteritidis outbreaks recognized by the German NRC in the past years were caused by a variety of comparatively rare phage types other than type PT 4, rendering phage typing by itself (as well as in combination with molecular methods) an excellent tool to pick up associations among cases, and between human infections and *S*. Enteritidis in foods.

The method of utilizing stored data on food orders instead of querying actual consumption introduces some uncertainty into exposure assessment. However, in circumstances such as these, with a population largely not competent to remember their exposure it may be a useful method to filter suspicious food items from a long list of potential exposures. In using the method, measures of association have to be interpreted with even more care than usual. Comparatively low RR in the context of infectious diseases, as well as borderline significant p values need to be evaluated in view of the imprecise exposure assessment. Analysis of food consumption ought to be complemented by food trace back and scrutiny of HACCP (Hazard Analysis Critical Control Point) protocol in order to stop current outbreaks and avoid repeated exposures.

## Conclusion

Nursing homes, with their population of vulnerable residents, are at a particular risk of *Salmonella *outbreaks, commonly caused by *S*. Enteritidis [8,9]. Baked goods, especially cakes containing non-heated components, are competent food vehicles for *Salmonella *outbreaks [10,11] and were the source of this large and protracted outbreak. Continuous low grade bacterial contamination of certain food items in combination with imperfect food handling practices can lead to protracted outbreaks of diarrhoeal disease in vulnerable populations – especially in summer when ambient temperatures facilitate bacterial proliferation in non-refrigerated foods.

Recommendations for nursing homes to avoid similar outbreaks are:

- to make sure baked goods subject to bacterial contamination are refrigerated until time of consumption, especially during the summer,

- to not delegate responsibility for refrigeration to residents, and

- to avoid serving certain "risky" foods like layered cream cake to those most at risk for serious disease consequences.

In addition, the following measures would have greatly enhanced outbreak detection and investigation in this outbreak, and appear useful to establish in similar institutions on a continuous basis:

- the storage of reference food samples covering ALL non-packaged food items served to the inhabitants, not only those prepared in the nursing home's kitchen, and

- strong infectious disease surveillance including the keeping of daily symptom logs, establishing the background rate of diarrhoeal symptoms (as well as respiratory symptoms) among residents of large facilities in order to more clearly identify the cessation of protracted outbreaks, and to promptly pick up outbreaks of norovirus, influenza etc. such that counter measures can be initiated without delay [12].

## Competing interests

The author(s) declare that they have no competing interests.

## Authors' contributions

CF and UB initiated the epidemiological investigation, designed the study, participated in the analysis and drafted the manuscript. MM assisted in the outbreak investigation, in the data collection, the epidemiological description and manuscript preparation. AS and KHB collected data on cases, information about exposure and risk factors of cases and non cases, implemented control measures and aided in writing the manuscript. PGD conducted the environmental investigations in the bakery implemented control measures and helped writing the manuscript.

WR conducted a large part of the laboratory investigation, participated in the analysis and helped to draft the manuscript. GF conducted major parts of the data analysis, wrote the formal final report of the investigation and helped to draft the manuscript. All authors read and approved the final manuscript.

## Pre-publication history

The pre-publication history for this paper can be accessed here:


